# Characterizing the Network of Drugs and Their Affected Metabolic Subpathways

**DOI:** 10.1371/journal.pone.0047326

**Published:** 2012-10-24

**Authors:** Chunquan Li, Desi Shang, Yan Wang, Jing Li, Junwei Han, Shuyuan Wang, Qianlan Yao, Yingying Wang, Yunpeng Zhang, Chunlong Zhang, Yanjun Xu, Wei Jiang, Xia Li

**Affiliations:** College of Bioinformatics Science and Technology, Harbin Medical University, Harbin, People’s Republic of China; Michigan State University, United States of America

## Abstract

A fundamental issue in biology and medicine is illustration of the overall drug impact which is always the consequence of changes in local regions of metabolic pathways (subpathways). To gain insights into the global relationship between drugs and their affected metabolic subpathways, we constructed a drug–metabolic subpathway network (DRSN). This network included 3925 significant drug–metabolic subpathway associations representing drug dual effects. Through analyses based on network biology, we found that if drugs were linked to the same subpathways in the DRSN, they tended to share the same indications and side effects. Furthermore, if drugs shared more subpathways, they tended to share more side effects. We then calculated the association score by integrating drug-affected subpathways and disease-related subpathways to quantify the extent of the associations between each drug class and disease class. The results showed some close drug–disease associations such as sex hormone drugs and cancer suggesting drug dual effects. Surprisingly, most drugs displayed close associations with their side effects rather than their indications. To further investigate the mechanism of drug dual effects, we classified all the subpathways in the DRSN into therapeutic and non-therapeutic subpathways representing drug therapeutic effects and side effects. Compared to drug side effects, the therapeutic effects tended to work through tissue-specific genes and these genes tend to be expressed in the adrenal gland, liver and kidney; while drug side effects always occurred in the liver, bone marrow and trachea. Taken together, the DRSN could provide great insights into understanding the global relationship between drugs and metabolic subpathways.

## Introduction

The increasing rate of failure in drug designs over the past few decades is mainly due to the dominant assumption which has historically relied upon particular families of “druggable” proteins [1]–[5]. More and more evidences has suggested that rational drug designs should focus on all the drug-affected proteins simultaneously and on the cellular networks or pathways formed by these proteins from a genome-wide perspective [4], [6], [7]. Metabolic pathways, which have crucial and broad functions in organism, participate in most processes of drug action on the cell and may be potentially available for drug discovery [8]–[13]. In particular, if a local region of a metabolic pathway (subpathway) contains many drug-affected proteins, this region may be highly associated with these drugs. The special regions containing highly-interactive proteins among the intricate metabolic pathways could help us understand the underlying mechanism of drug action on a subtle level [14], [15]. Moreover, different drugs often exert both therapeutic and adverse effects, because the proteins affected by these drugs may interact with each other in a metabolic subpathway to carry out special biological function together [16], [17]. In addition, a drug may affect many metabolic subpathways simultaneously carrying therapeutic or adverse effects. Based on the complex relationships between drugs and their affected metabolic subpathways, a large-scale drug–subpathway network should be established and network analysis should be utilized to study drug action on these metabolic subpathways which could provide a system-level understanding of drug action [18]–[20]. A variety of studies have demonstrated the power of network analysis on biology and pharmacology [1], [2], [4], [5], [16], [21]–[23]. However, it is difficult to construct the global relationship between drugs and metabolic subpathways using traditional biological experimental studies. Furthermore, many studies have focused on one aspect of drug impact (for example, drug targets or side effects) and not take into account the global relationships between drugs and the affected metabolic subpathways [5], [21], [24], [25].

These limitations could be alleviated to a great extent by the development of high-throughput experimental and bioinformatics technologies. On the one hand, some databases such as the Connectivity Map (CMap), which is a genome-wide transcriptional expression data set of selected human cells (5 cell lines) treated with bioactive small molecules including many drugs [26], could provide a system detailing the impact of drugs including therapeutic and side effects. On the other hand, high quality pathway structure data from the Kyoto Encyclopedia of Genes and Genomes (KEGG) and some software packages are available for effectively identifying drug-affected regions of metabolic pathways [17], [27]. Thus, we can obtain the drug-affected genes from expression profiles in CMap and then identify corresponding subpathways by the pathway-enriched method. Here, we generated a bipartite drug metabolic subpathway associated network (DRSN) in which nodes represent drugs and metabolic subpathways and they were connected if the drug-affected genes could be significantly enriched to the subpathways. We then combined network biology and pharmacology to (i) analyze the relationships between drug dual effects and metabolic subpathways based on the connection in the DRSN, (ii) explore the global inter-relationships between diseases and drug response through metabolic subpathways, and (iii) assess the tissue-specific differences between drug therapeutic and non-therapeutic subpathways. Our results showed that the DRSN may not only offer insights into understanding underlying mechanisms of drug actions but also provide a rational way to improve the drug efficacy and clinical safety.

## Results

### Generating the Drug–metabolic Subpathway Network

**Figure 1:**
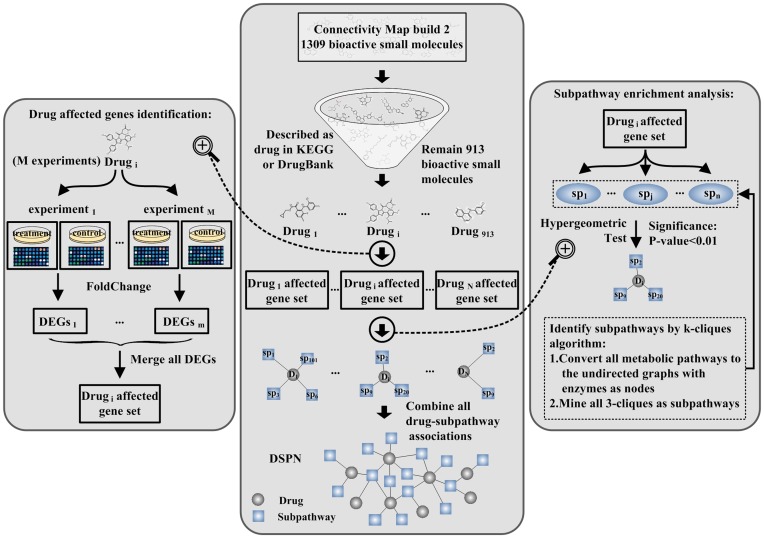
Schematic of the construction of the drug–metabolic subpathway network. We generated the drug–metabolic subpathway network (DRSN) based on the drug-affected genes and pathway structure data from KEGG. First, we selected the small molecules which were used as drugs and then found the affected genes for each drug from corresponding expression profiles in CMap. Second, for each drug, we applied the “k-clique” subpathway identification method to identify the statistically significantly enriched subpathways by setting k = 3 and P-value <0.01 to obtain associations between the drugs and metabolic subpathways. Finally, we combined these drug–metabolic subpathway associations to form the DRSN.

**Figure 2:**
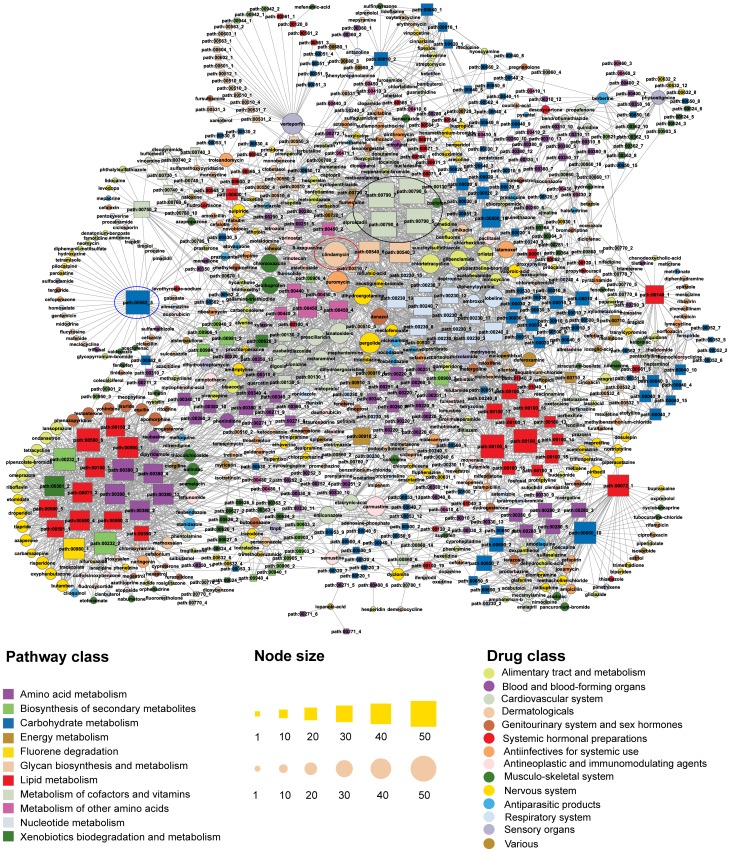
The DRSN network. The circles and rectangles in the network correspond to drugs and metabolic subpathways, respectively. A drug and a metabolic subpathway are connected by an edge if the corresponding drug-affected genes are statistically significantly enriched to the corresponding subpathway. Node size is proportional to the degree of the node. Nodes are colored according to 14 ATC and 11 KEGG pathway categories.

We used genome-wide transcriptional expression data from the CMap database and pathway data from the KEGG database to construct the drug–metabolic subpathway network (DRSN) (Figure 1). Currently, CMap contains 1309 bioactive small molecules corresponding to 6100 instances (experiments) [26]. First, we selected the bioactive small molecules which were used as drugs according to drug type descriptions extracted from the DrugBank and the KEGG DRUG database [27], [28]. After some automatically and manually dealing steps such as matching bioactive small molecule names to drug names and adding drug classifications, etc. (see Text S1), we obtained 913 small molecular drugs which were grouped into 14 drug classes using the Anatomical Therapeutic Chemical (ATC) classification system (see Dataset S1). Every drug (bioactive small molecule) has several perturbation experiments (instances) under different conditions. Second, we used fold-change analysis to identify differentially expressed genes (DEGs) for every instance with |log2fold change|>1 from the corresponding treatment and control gene expression profiles. The DEGs were merged if the corresponding experiments belonged to the same drug and these genes were considered to be affected genes for this drug. After the above steps, we obtained 128734 unique drug–gene associations composed of 10412 drug-affected genes and 913 drugs. Third, we used the “k-clique” subpathway identification method from the SubpathwayMiner software package to identify significantly drug affected metabolic subpathways [17] (see Text S1). There were 743 subpathways when k = 3. This parameter setting meant that the distance among the enzymes in one subpathway was not greater than 3 to ensure that enzymes in the subpathways have highly similar functions. Finally, for each of the 913 drugs, we used enrichment analysis in the “k-clique” subpathway identification method to identify the statistically significant drug-affected subpathways based on the corresponding drug-affected gene set with a P-value<0.01. We then found that 488 drugs were significantly associated with 403 subpathways. These drugs and subpathways generated 3925 significant associations (see Dataset S2). We then constructed a bipartite drug–metabolic subpathway network consisting of small molecular drugs and drug-related metabolic subpathways in which a drug and a subpathway were connected if the drug-affected genes could be significantly enriched in the corresponding subpathways (Figure 2).

### The Basic Properties of the DRSN

The DRSN was composed of 891 nodes (403 subpathways and 488 drugs), and 3925 edges (Figure 2). 883 nodes formed a giant component suggesting that the drugs and metabolic subpathways were closely connected in the DRSN. The average degree of drug and subpathway nodes in the DRSN was significantly higher than that of 1000 randomized networks (P-value<0.001, see Figure S1A and S1B) and the edges in the DRSN were denser than that in randomized networks (P-value = 0 see Figure S1C), suggesting that the drugs and metabolic subpathways were closely connected at the system level. The reason for this may be that the DRSN could represent the dual and complicated relationships between drugs and subpathways. The degree of distribution of the drug and subpathway nodes both followed power law distributions approximately with a slope of −0.5835 and −0.887 respectively and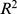 = ∼0.9421 and ∼0.966 respectively (Figure S2A and S2B). Thus, the DRSN was scale-free [29]. These results suggested that a few subpathway nodes linked many drugs and a few drug nodes act as hubs with a large number of links to subpathway nodes.

**Figure 3:**
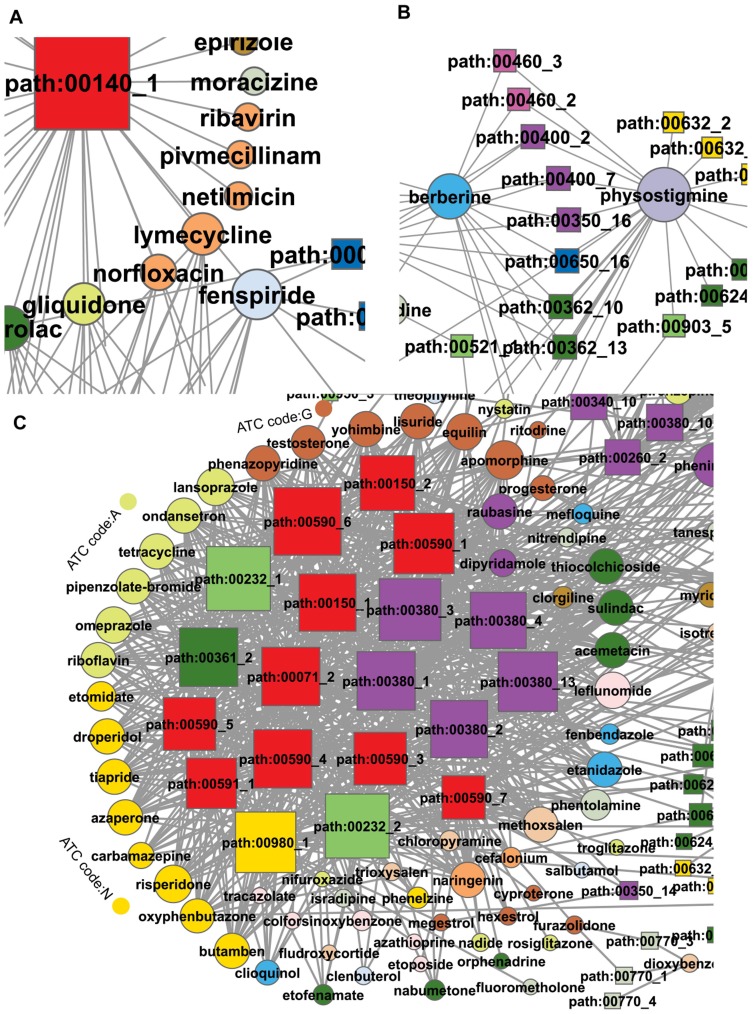
Three examples representing features of DRSN. All the examples are from the DRSN (**A**) Some antibiotic drugs were linked to steroid hormone biosynthesis (path:00140_1). (B) Berberine and physostigmine were linked to the same subpathways. (C) Some drugs and subpathways were closely connected closely in the DRSN. The drugs mainly belonged to alimentary tract and metabolism, nervous system, and genitourinary system and sex hormones classes. The subpathways mainly belonged to three pathways: androgen and estrogen metabolism (path:00150), tryptophan metabolism (path:00380) and arachidonic acid metabolism (path:00590).

The degree of subpathway nodes spanned a wide range from 1 to 53. Considering the top five highest degree subpathways nodes, four nodes with degree>50 belonged to the folate biosynthesis pathway (path:00790) (black ellipse in Figure 2), which is important in drug design and is the potential therapeutic targets of many types of drugs including antibiotics and anticancer drugs, etc. [30]–[33]. Another subpathway node in the top five was inositol phosphate metabolism (path:00562_5) (degree = 48) (blue ellipse in Figure 2). Defects in this pathway contribute to many diseases including cancers, leukemias, immunodeficiencies, autoimmune, neurodegenerative, allergic and inflammatory disorders [34]. Some reports have indicated that many drugs such as anti-bipolar, anticancer, cardiovascular and anti-inflammatory drugs could target the inositol phosphate metabolism pathway [35]–[38]. In contrast, many subpathways with a low degree were connected to only a few drugs. For example, some subpathways belonging to glycan biosynthesis and metabolism were only linked to verteporfin. Some subpathways in the DRSN revealed the potentially functional mechanisms of drug side effects. For example, as shown in Figure 3A, some antibiotic drugs such as ribavirin were linked to steroid hormone biosynthesis (path:00140_1). Studies showed that antibiotic treatment could affect normal steroid hormone synthesis and lead to a disrupted intestinal homeostasis [39].

Similar to subpathway nodes, the drug nodes also displayed evident differences in connection. The highest degree drug node (degree = 53) was clindamycin (red ellipse in Figure 2), an antibiotic, which can be used in topical or systemic treatment [40]. It was linked to many types of subpathways, partly because of its broadly large effects [41]. On the one hand, clindamycin can cause a hypersensitivity reaction in many tissues including colon, vascular, sensory organs, muscle, epithelium and liver [42]–[47]. On the other hand, there may be many unknown clindamycin-induced therapeutic and side effects which could provide insights into drug repositioning. To alleviate the influence of chosen threshold of hypergeometric tests, we also constructed a DRSN with P-value = 0.05. We found that clindamycin had second highest degree (degree = 104). Many drug nodes with degree = 1 were observed in the DRSN. For example, hesperidin, a vitamin with anti-carcinogenic activity against lung cancer [48], was only linked to phenylalanine, tyrosine and tryptophan biosynthesis (path:00400_6). Some studies have reported that the concentrations of tyrosine and tryptophan were changed in lung cancer [49], suggesting a potential new anticancer mechanism of hesperidin. Interestingly, some drugs belonging to different ATC classes were connected to the same subpathways in the DRSN. A possible reason for this is that these drugs may affect the same biological process which may not have been noticed before. As an example, berberine and physostigmine, which belong to different ATC classes, were connected to the same subpathways (Figure 3B and Table 1). Studies have shown that both these drugs can inhibit acetylcholinesterase activity [50]. To better interpret drug–subpathway associations, we searched more literatures about some drug–subpathway associations from various significant levels (Table S1).

**Table 1:** Information of example subpathways in Figure 3.

	SubpathwayID	Pathway name
**Example 1**	path:00140_1	C21-Steroid hormone metabolism
**Example 2**	path:00460_2	Cyanoamino acid metabolism
	path:00460_3	Cyanoamino acid metabolism
	path:00400_2	Phenylalanine, tyrosine and tryptophan biosynthesis
	path:00400_7	Phenylalanine, tyrosine and tryptophan biosynthesis
	path:00350_16	Tyrosine metabolism
	path:00650_16	Butanoate metabolism
	path:00362_10	Benzoate degradation via hydroxylation
	path:00362_13	Benzoate degradation via hydroxylation
**Example 3**	path:00590_1	Arachidonic acid metabolism
	path:00590_3	Arachidonic acid metabolism
	path:00590_4	Arachidonic acid metabolism
	path:00590_5	Arachidonic acid metabolism
	path:00590_6	Arachidonic acid metabolism
	path:00590_7	Arachidonic acid metabolism
	path:00150_1	Androgen and estrogen metabolism
	path:00150_2	Androgen and estrogen metabolism
	path:00071_2	Fatty acid metabolism
	path:00591_1	Linoleic acid metabolism
	path:00232_1	Caffeine metabolism
	path:00232_2	Caffeine metabolism
	path:00380_1	Tryptophan metabolism
	path:00380_2	Tryptophan metabolism
	path:00380_3	Tryptophan metabolism
	path:00380_4	Tryptophan metabolism
	path:00380_13	Tryptophan metabolism

The ID and names of example subpathways are provided. These subpathways were used as examples to illustrate the DRSN. Example 1, 2, 3 correspond to Figure 3A, 3B and 3C, respectively.

It was noted that some drugs and subpathway nodes were connected more closely and formed a module in the DRSN. As shown in Figure 3C, these drugs mainly belonged to the alimentary tract and metabolism (ATC code: A), nervous system (ATC code: N), and genitourinary system and sex hormones (ATC code: G) class. The subpathways mainly belonged to three pathways: androgen and estrogen metabolism, tryptophan metabolism and arachidonic acid metabolism (Table 1). The connections between these drugs and the subpathways were potentially valuable. On the one hand, some connections between drugs and subpathways revealed drug therapeutic effects. For example, some sex hormone drugs were linked to androgen and estrogen metabolism; and some nervous system drugs were connected to tryptophan metabolism, which is considered to be involved in serotonergic neuronal function [51], [52]. Also, some connections between drugs and subpathways provided potentially novel therapeutic targets and applications for existing drugs. For example, in Figure 3C, arachidonic acid metabolism subpathways were connected to three classes of drugs. Some studies have suggested that sex hormones such as testosterone and progesterone, and some metabolism drugs and nervous system drugs can modulate arachidonic acid metabolism in the brain or in neural membranes and the arachidonic acid metabolism pathway is a potential therapeutic target for neurological diseases [53]–[59]. On the other hand, some connections revealed the mechanisms of potential drug side effects. For example, the metabolism and nervous system drugs were connected to androgen and estrogen metabolism subpathways. Studies have shown that omeprazole and some nervous system drugs are possibly agents responsible for gynecomastia due to an impaired balance in the serum estrogen/serum androgen ratio [60].

### Drug Therapeutic and Side Effects and Metabolic Subpathways in the DRSN

Drugs can produce desirable and undesirable changes in the physiological cellular process and lead to subsequent therapeutic effects and side effects. In the DRSN, the connections between drugs and subpathways revealed the drug dual effects on the human body. Some researchers have indicated that drugs binding to similar proteins tend to cause similar effects and these affected proteins may interact with each other to form a biological subpathway [21], [61]–[63]. Thus, if two drugs were connected to the same subpathways in our network, they were likely to cause the same therapeutic or side effects.

**Figure 4:**
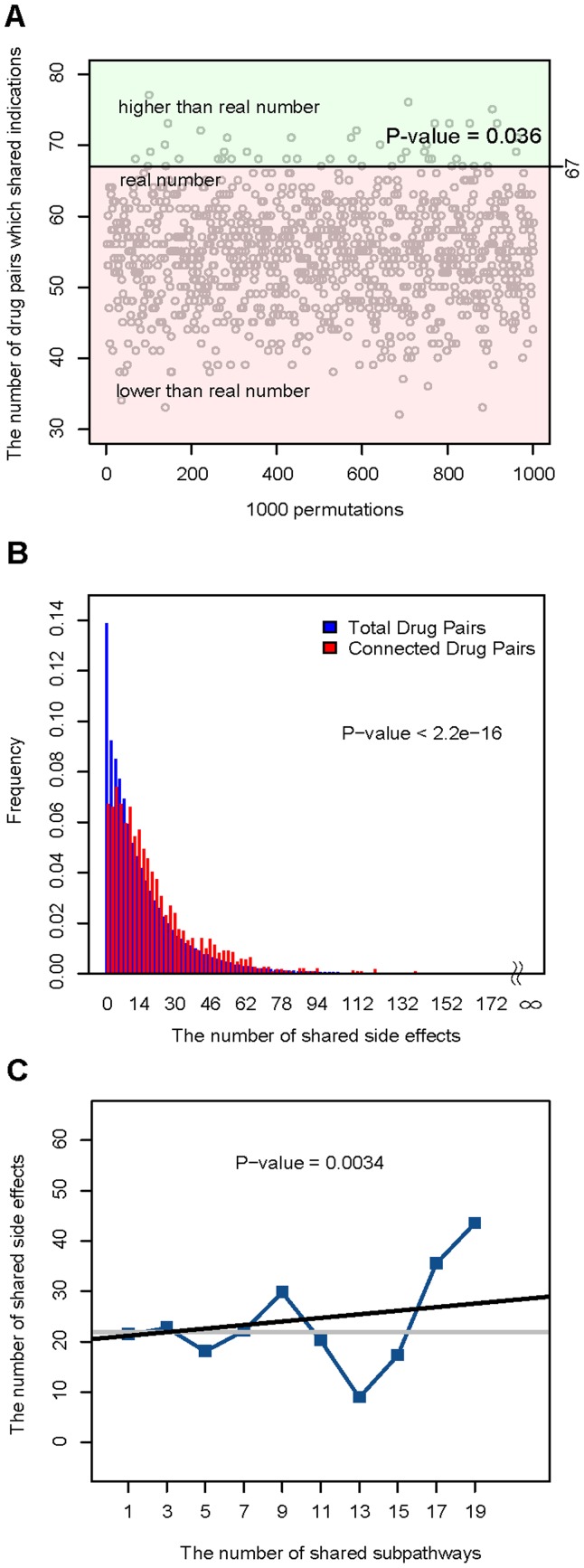
The relationship between drug dual effects and metabolic subpathways in the DRSN. (A) Of 1586 connected drug pairs (drugs that were linked to the same subpathways), 67 shared the same indications, compared to 55 drug pairs on average of 1000 randomized 1586 drug pairs. Of 1000 times randomized 1586 drug pairs, there were only 36 times when the number of drug pairs which shared the same indications were more than 67 (P-value = 0.036). (B) The number of side effects shared by connected drug pairs was significantly higher than the number of side effects shared by all drug pairs in the SIDER database (P-value <10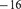). (C) The number of shared side effects significantly increased as the number of the shared subpathways increased between two drugs (P-value = 0.0034). The grey horizontal line is the average number of side-effects all drug pairs shared. The Y axis represents the number of side-effects shared by drug pairs. The X axis represents the number of the same subpathways drug pairs shared. Blue “▪” symbols correspond to the binned average number of side-effects shared by drug pairs. The linear regression model (black line) is used to test the trends in correlations and the significance of the trends is estimated.

To examine this, we first downloaded the dataset of drug–indication association from the paper by Yildirim et al. (see Materials and methods) [5]. In the DRSN, there were 160 drugs recorded in this drug–indication association and these drugs formed 1586 unique connected drug pairs (two drugs which were connected by the same subpathways in the DRSN). Of these connected drug pairs, 67 shared the same indications. We then generated 1586 randomized drug pairs for 1000 times. We found that there were only 36 times when the number of randomized drug pairs which shared the same indications were more than 67, suggesting that connected pairs tended to share the same indications (P-value = 0.036) (Figure 4A). We then downloaded the public and accurate side-effect records from the SIDER database including 888 drugs corresponding to 1450 side effects [64]. In the DRSN, there were 199 drugs recorded in the SIDER database and these drugs formed 2350 unique connected drug pairs. In the SIDER database, some side effects, such as dizziness and nausea, were caused by most drugs [21]. To improve the specificity of similarity of drug pairs, we calculated the number of side effects shared by drug pairs rather than the number of drug pairs which shared the same side effects. We found that the number of side effects shared by connected drug pairs was also significantly higher than the number of side effects shared by total drug pairs in the SIDER database (P-value<10
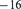
Wilcoxon rank-sum test) (Figure 4B). These results suggested that two drugs connected by the same subpathways in the DRSN tended to be used for the same indications and cause the same side effects. Thus, the DRSN may help us explain the mechanism of drug therapeutic effects and unwanted toxicity caused by drugs. As shown in Figure 3C, some sex hormone drugs were connected to subpathways involved in androgen and estrogen metabolism (path:00150), suggesting the therapeutic effects of these sex hormone drugs. Also, in Figure 3A, some antibiotics such as netilmicin and lymecycline were connected to steroid hormone biosynthesis (path:00140_1). This subpathway is related to disrupted intestinal homeostasis which is a side effect always caused by antibiotics [39].

In the DRSN, we found that the number of subpathways shared by drug pairs ranged from 1 to 24. Thus, we questioned whether more subpathways shared by two drugs mean more shared side effects. Furthermore, we measured the correlation between the number of the subpathways and the number of side effects shared by the connected drug pairs, and found that the number of shared side effects significantly increased as the number of the shared subpathways increased (P-value = 0.0034) (Figure 4C). These results indicated that if two drugs affected more subpathways together, they tended to cause more of the same side effects.

### Exploring Drug–disease Associations Through Metabolic Subpathways

**Figure 5:**
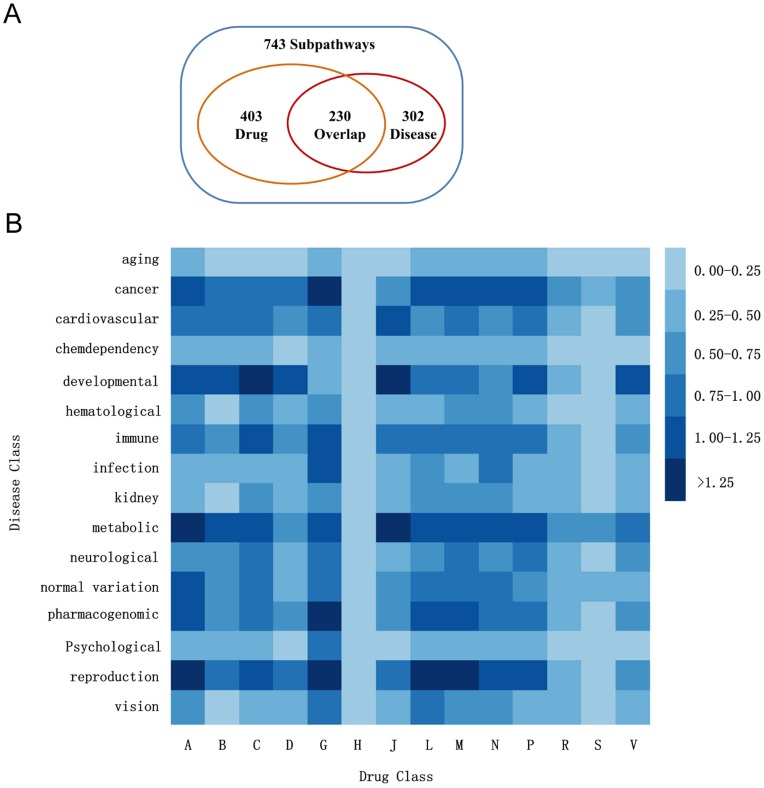
Drug–disease associations through affected metabolic subpathways. (A) There were 743 metabolic subpathways when the setting k = 3. There were 302 disease–related subpathways according to the disease–metabolic subpathways association in the DMSPN and 403 drug-affected subpathways in the DRSN. 230 subpathways were overlapped between the two sets of subpathways. (B) The association scores between each drug class and disease class. The darker color represents the higher association scores.

In the DRSN, the drug-affected subpathways revealed the molecular mechanism of drug therapeutic effects and side effects. Abnormalities in the biological functions of subpathways are highly associated with the initiation and progression of many diseases. It is likely that on the one hand, drugs exerted their therapeutic effects through the subpathways related to the corresponding indications; on the other hand, drugs also caused many undesirable diseases (side effects) through the corresponding disease-related subpathways. Thus, we can elucidate the dual relationships between drugs and diseases through the metabolic subpathways. To do this, we obtained the disease-metabolic subpathways associations from the disease–metabolic subpathway network (DMSPN) in our previous work [65] (see Materials and methods). There were 545 nodes (302 subpathways and 243 diseases) in the DMSPN, of which 230 subpathway nodes were in the drug–metabolic subpathway network. This indicated that a large proportion (76%) of disease-related metabolic subpathways was also affected by drugs (Figure 5A). We calculated the association scores (see Materials and methods and Text S1) between each drug class and each disease class (Figure 5B). The association scores quantified the extent of correlation between each drug class and disease class by the shared subpathways. As shown in Figure 5B, we found that a few drug classes displayed high correlation with their corresponding indications, especially “alimentary tract and metabolism” drugs (ATC code: A) and metabolic diseases. Another example was the genitourinary system and sex hormones drugs (ATC code: G) and reproductive diseases. This indicated that some drugs exert their therapeutic effects through the subpathways related to the corresponding indications. However, we found that most drug classes were highly associated with other disease classes rather than their indications suggesting that the drugs could cause these undesirable diseases (side effects). For example, the “system anti-infective” drugs (ATC code: J) displayed close correlations with development diseases and metabolic diseases suggesting that the system anti-infective drugs may potentially lead to side effects belonging to these disease classes. Furthermore, we also found some interesting correlations between drugs and diseases in Figure 5B, representing dual and complicated drug-disease relations. For example, “genitourinary system and sex hormone” drugs (ATC code G) displayed a high correlation with cancers. Some studies have shown that sex hormones have played a dual and implicated role in the progression of breast, prostate, gynecologic, and colorectal cancer [66]. However, the detailed mechanism is still unclear. A possible reason for this phenomenon is that some sex hormone drugs affected the subpathways belonging to tryptophan metabolism (path:00380), arachidonic acid metabolism (path:00590) and caffeine metabolism (path:00232) (Figure 3C). These subpathways were also highly correlated with cancers in the disease–metabolic subpathway network [65]. These results might provide insights into cancer therapy and prevention.

On the contrary, some drugs displayed low correlations with almost all disease classes (for example, “systemic hormonal preparations” drugs and “sensory organs” drugs corresponding to ATC code: H and S). These drugs may cause fewer side effects which were spread rather than centered in one disease class. Similarly, some disease classes also showed low correlation with most drug classes (For example, aging diseases). The reason for this may be that there were few drugs able to treat these diseases and most drugs did not lead to undesirable diseases (side effects).

### Tissue-specificity Analysis of Drug Therapeutic Effects and Side Effects Through Subpathways

**Figure 6:**
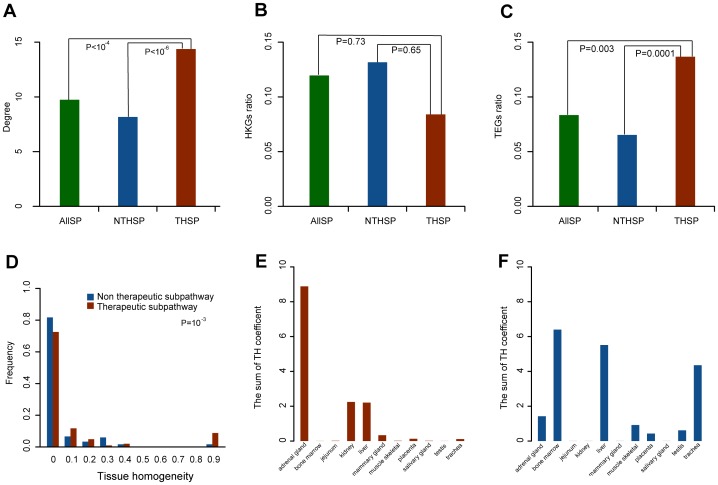
Tissue-specific differences between therapeutic and non-therapeutic subpathways Therapeutic and non-therapeutic subpathways had tissue-specific differences. (**A**) The degree of therapeutic subpathways (THSP) was significantly higher than that of non-therapeutic subpathways (NTHSP) (P-value <10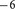) and all subpathways (ALLSP) (P-value <10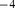). (B) There were no significant differences in the ratios of drug-affected HKGs between the two types of subpathway (P-value = 0.65) as well as that between therapeutic subpathways and all subpathways (P-value = 0.73). (C) The ratios of drug-affected TEGs in drug therapeutic subpathways (average ratio = 0.14) were higher than that of non-therapeutic subpathways (average ratio = 0.065; P-value = 0.0001) and that of all subpathways (average ratio = 0.08; P-value = 0.003) (D) TH coefficients of drug therapeutic subpathway were significant higher than that of non-therapeutics (P = 0.0001) (E) The sum of TH coefficients of drug therapeutic subpathways in different tissues (F) The sum of TH coefficients of drug non-therapeutic subpathways in different tissues.

Drug therapeutic effects and side effects always occur in different tissues because they are carried out by different genes which are expressed in different tissues and make a desirable or undesirable change in different physiological cellular pathways [62]. To examine the tissue specificity of drug therapeutic effects and side effects, we classified all the subpathways in the DRSN into therapeutic subpathways and non-therapeutic subpathways according to whether they had therapeutic targets in the drug-affected genes. Of the 488 drugs in the DRSN, 293 drugs have 325 therapeutic targets (see Text S1 and Dataset S3). If the drug-affected genes in a subpathway contained these therapeutic targets, this subpathway was considered to be a drug therapeutic subpathway corresponding to drug therapeutic effects. Otherwise it was considered as non-therapeutic subpathway involved in drug side effects. We thus classified all 403 metabolic subpathways into 102 therapeutic subpathways and 301 non-therapeutic subpathways. We found that the degree of therapeutic subpathways was significantly higher than that of non-therapeutic subpathways (P-value<10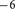) and all the subpathways (P-value<10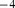) (Wilcoxon rank-sum test) (Figure 6A). These findings suggested that the therapeutic subpathways were deliberately prone to be affected by drugs for easy implementation of drug therapeutic effects in patients.

The therapeutic and non-therapeutic subpathways represented drug therapeutic effects and side effects respectively. The tissue-specific expression of drug-affected genes in the subpathways was responsible for the tissue specificity of drug therapeutic effects and side effects. To test this, we obtained the housekeeping genes (HKGs) and tissue-specific genes (TEGs) dataset from the paper by She et al. The dataset contained the gene expression profiles of 42 normal human tissues and identified 1,522 HKGs and 975 TEGs [67]. We then examined the ratios of the number of TEGs and HKGs to the number of all the drug-affected genes in each therapeutic/non-therapeutic subpathway in the DRSN. We found that there were no significant differences in the ratios of drug-affected HKGs between the two types of subpathway (P-value = 0.65) and between the therapeutic subpathways and all subpathways (P-value = 0.73) (Wilcoxon rank-sum test) (Figure 6B). However, the average ratio of drug-affected TEGs in drug therapeutic subpathways was 0.14, nearly one-fold larger than that of non-therapeutic subpathways (average ratio = 0.065) (P-value = 0.0001) and that of all subpathways (average ratio = 0.08) (P-value = 0.003) (Wilcoxon rank-sum test) (Figure 6C). This showed that HKGs were expressed more stably than TEGs [68] and maintained a stable ratio in drug-affected genes in all types of subpathways. However, the TEGs tended to represent different physiological processes and were considered as candidates for drug therapeutic targets [67]. Our finding indicated that the therapeutic subpathways were more likely to be activated in different tissues by drugs, thus the drug therapeutic effects displayed a tissue-specific tendency.

Although therapeutic subpathways had higher drug-affected TEGs ratios than non-therapeutic subpathways, we attempted to assess whether the TEGs in a subpathway tended to be expressed in one tissue. To test this, we calculated the tissue-homogeneity (TH) coefficient for a subpathway. The TH coefficient introduced by Goh et.al. [69] is defined here as the maximum fraction of genes which were expressed in a specific tissue among all drug-affected TEGs belonging to one subpathway (see Materials and methods). The TH coefficient quantified whether drug-affected TEGs in one subpathway tended to be expressed in one tissue. If all drug-affected TEGs in a subpathway were expressed in one tissue, the TH coefficient was 1 representing perfect tissue-homogeneity in this subpathway. We found that the TH coefficients of drug therapeutic subpathways were significantly higher than that of non-therapeutic subpathways (P = 0.0001 using Wilcoxon rank-sum test), implying that drug-affected TEGs in therapeutic subpathways tended to be expressed in fewer tissues than those in non-therapeutic subpathways (Figure 6D). A potential explanation for this is that therapeutic subpathways should be deliberately under-targeted in other tissues, except the target tissues, to avoid potential side effects [70].

For each subpathway, the TH coefficient was identified as the maximum fraction of genes which were expressed in a specific tissue among all drug-affected TEGs in this subpathway. The specific tissue in which most drug-affected TEGs were expressed was considered as the most affected tissue for a subpathway. We calculated the sum of the TH coefficient for each tissue for therapeutic and non-therapeutic subpathways respectively to examine the difference in affected tissues between drug therapeutic effects and side effects. Compared to non-therapeutic subpathways, in therapeutic subpathways, three tissues (adrenal gland, liver and kidney) had a high sum of TH coefficient (Figure 6E), suggesting that these tissues contained more therapeutic targets. For example, the adrenal gland, an important endocrine tissue, is the target of a large number of exogenous chemicals including many drugs [71]. For the non-therapeutic subpathways, the sum of the TH coefficient for liver, bone marrow and trachea was higher than that for the therapeutic subpathways (Figure 6F). Many frequent side effects (asthma and anemia for example) were produced in bone marrow and trachea. For example, in the DRSN, some bone marrow specific non-therapeutic subpathways were connected to antazoline, a histamine receptor antagonist [72], which can induce immune hemolytic anemia, thrombocytopenia, and hemoglobinuria [73]. Another example was etodolac, a highly lipophilic anti-inflammatory drug [74], which was connected to trachea-specific non-therapeutic subpathways in the DRSN, and has been proved to have an adverse effect on the trachea and leads to asthma [75]. Interestingly, both drug therapeutic and side effects had an impact on the liver. The critical synthetic, metabolic, and detoxifying function of the liver may partly explain its therapeutic importance and arguably its vulnerability to be injured by drugs [76]. In summary, therapeutic subpathways and non-therapeutic subpathways had significant tissue specific differences including the ratio of TEGs and HKGs in drug-affected genes, the TH coefficient and affected tissues. Therapeutic subpathways tend to be more tissue-specific and non-therapeutic subpathways have specific functional tissues for drug degradation and metabolism.

## Discussion

We constructed a drug–metabolic subpathway network (DRSN) based on drug-affected genes using the “k-clique” subpathway identification method. To date, many studies on drugs and their affected subpathways have been limited to low-throughput experiments and many large scale studies on drugs have only focused on one aspect of drug impact (therapeutic effects or side effects) [5], [21], [24], [25]. These limitations hinder the assessment of cellular processes under drug influence to a great extent and do not provide a global insight into rational drug design. In our study, the drug-affected genes were obtained from whole genome gene expression profile data in the CMap database, thus providing a resource of genome-wide drug influence on the human body including therapeutic effects and side effects. We used fold change analysis to identify drug-affected genes with |log2fold change|>1, which could control the quality to some extent. Furthermore, by applying the “k-clique” subpathway identification method to all drug-affected gene sets, we identified affected subpathways for all drugs and showed that on the one hand, the genes in one subpathway were highly interactive and tended to be involved in similar biological processes; on the other hand, the affected subpathways indicated the overall impact of drugs on the human body. We used the hypergeometric test in “k-clique” subpathway identification method to identify drug-affected subpathways. Hypergeometric test accesses the enrichment significance of a gene set rather than individual genes. Individual noise would not have a significant influence on the enrichment accuracy. In particular, we selected a strict significance threshold of 0.01. These measures further alleviated the noise to a great extent. We finally obtained 3925 drug–metabolic subpathway associations composed of 488 drugs and 403 metabolic subpathways. Taking the drug–metabolic subpathway associations together, we were able to investigate the global relationship between drugs and their affected subpathways.

In the DRSN, some drug–metabolic subpathway connections revealed the mechanism of drug therapeutic effects such as links between sex hormone drugs and androgen and estrogen metabolism, while some connections revealed side effects such as links between and links between antibiotic drugs and steroid hormone biosynthesis. Interestingly, some connections suggested potentially novel therapeutic targets and applications such as links between arachidonic acid metabolism and three classes of drugs (Figure 3C). Furthermore, many drugs which belonged to different classes were connected to the same metabolic subpathways (Figure 3B as an example). These new drug–drug relationships might offer insights into drug combinations and drug repositioning. We also found that if drugs were connected to the same subpathways in the DRSN, they tended to share the same therapeutic effects and side effects and the number of shared side effects increased as the number of shared subpathways increased. These results showed that the drug-affected subpathways in the DRSN could provide an in-depth understanding of the molecular mechanism of drug therapeutic and side effects. We further investigated the drug–disease associations by integrating drug-affected subpathways in the DRSN and disease-related subpathways. After calculating the association scores between each drug class and disease class, we found that most drug classes were closely related to their side effects rather than their indications. It is worth noting that our work also suggested drug dual effects. For example, some sex hormone drugs displayed a high correlation with cancers. These drug–disease associations through the subpathways may help us find new indications for existing drugs and identify novel side effects. Finally, to further study the mechanism of drug dual effects, we classified all the subpathways in the DRSN into therapeutic and non-therapeutic subpathways representing drug therapeutic effects and side effects, respectively. Compared to side effects, the therapeutic effects tended to affect more tissue-specific genes and these genes tended to be expressed in fewer tissues, indicating that drug therapeutic effects should be deliberately under-targeted in other tissues, except its target tissues, to avoid potential side effects. After calculating the sum of the TH coefficient for all the tissues, we found that the therapeutic effects tended to affect the tissues such as the adrenal gland which is important in drug therapy, while side effects always affected tissues such as liver which is vulnerable to injury by drugs. The research on tissue-specific differences between two kinds of subpathways could improve our understanding of cellular function in different tissues due to drug action as well as the mechanism of drug therapeutic and side effects. Taken together, the DRSN offered a comprehensive and functional understanding of the global relationship between drugs and metabolic subpathways.

To confirm the validity of our results, we also constructed the DRSN with k = 4 and repeated some of the analyses for the network. We found that the two networks and results of the two networks were robust (see Text S1, Figures S3 and S4). To alleviate the influence of chosen threshold of hypergeometric tests, we also constructed a DRSN with P-value = 0.05. We found that clindamycin had second highest degree (degree = 104). Furthermore, after we compared the drug degree rank of two networks, we found that there were 10 same drugs in the top 12 degree rank (see red font in Dataset S5). This result indicated that our method was robust in different threshold of hypergeometric tests. We also noted that there were several limitations in our study. Firstly, only small molecular drugs were included in the DRSN. The completeness of the DRSN will be improved by adding other types of drugs and integrating more drug-affected gene expression profiles from different resources. Another limitation of our DRSN is the incompleteness of the metabolic pathway data and the false positive results of the enrichment analysis. These limitations will be alleviated, to a great extent, with the development of a drug database and accurate reconstructions of metabolic networks and the integration of different pathway databases. We used subpathwayMiner to identify subpathways as the k-cliques method has been proved to be effective in subpathway identification [65], [77], [78]. We selected a relatively small distance k = 3 and 4 to ensure that enzymes in the subpathways have highly similar functions. This method could successfully identify the significant subpathways which may be ignored using the entire pathway identification method [17]. Although this method can more accurately identify drug-affected subpathway regions in pathways, further improvement of the identification strategy is also needed. For example, other factors such as linear/non-linear and hub genes, etc are likely to further improve accuracy of subpathway identification. Although our data and methodology are far from completeness, our analysis of the DRSN, based on the network pharmacology [4], [13], [79], [80], offers a comprehensive picture of global and significant associations between drugs and their subpathways (see Dataset S2) by considering the system effects of drugs at a functional level. A user-friendly web server, called the DRSN database, to query, visualize and download for all the data in our research can be freely accessed at http://bioinfo.hrbmu.edu.cn/DRSN.

## Materials and Methods

### Drug Database

We selected bioactive small molecules which are used as drugs according to the information from the DrugBank and KEGG DRUG database. As of vision 2.5, the DrugBank database contained nearly 4,800 drug entries, including >1,350 FDA-approved small molecule drugs, 123 FDA-approved biotech drugs, 71 nutraceuticals and more than 3,243 experimental drugs [28]. We extracted all the small molecule drugs according to the drug descriptions. For KEGG DRUG, we downloaded the drug card from website (ftp://ftp.genome.jp/pub/kegg/medicus/drug/) [27], and then extracted all the small molecule drugs. Of the 1309 small molecules in the Connectivity Map (CMap), 581 of these small molecules were recorded as drugs in DrugBank. Of the remaining 728 small molecules, 332 were recorded as drugs in KEGG DRUG. In CMap, we finally obtained 913 small molecular drugs (see Dataset S1) corresponding to 4320 instances (experiments).

For the 488 drugs in the DRSN, we extracted the therapeutic targets of these drugs from information in DrugBank and KEGG DRUG. We finally obtained 325 therapeutic targets corresponding to 293 drugs (see Dataset S3).

### CMap Database

We obtained the drug-affected genes from the Connectivity Map (CMap). In CMap, the genome-wide transcriptional expression data is from cultured human cells lines treated with bioactive small molecules [26]. To date, CMap contains 6100 instances corresponding to 1309 bioactive small molecules. We downloaded all the expression profiles and their associated annotation file “cmap_instances_02.xls” from the CMap website (http://www.broadinstitute.org/cmap/). We matched perturbation and control pairs of expression profiles for each instance according to descriptions of the instances in the file “cmap_instances_02.xls”. Then we used fold-change analysis to identify differentially expressed genes (DEGs) for each instance with |fold change|>1 from the corresponding treatment and control gene expression profiles. The DEGs were merged if the corresponding instances (experiments) belonged to the same drug (bioactive small molecule) and these genes were considered to be affected genes for this drug.

### SubpathwayMiner and Disease–metabolic Subpathway Network (DMSPN)

In this study, we used the “k-clique” subpathway identification method provided by the SubpathwayMiner software package [17] to identify statistically significantly enriched drug-affected subpathways. This method was developed according to pathway structure data provided by KEGG (see Text S1). After inputting gene sets (drug-affected genes) and distance parameter k, the method can mine each metabolic subpathways and then identify statistically significantly enriched subpathways with a P-value<0.01. By applying this method to all the drugs, we obtained the global relationships between drugs and metabolic subpathways.

We obtained the relationships between diseases and metabolic subpathways from the DMSPN which was constructed based on disease genes from the Genetic Association Database (GAD) and pathway structure data from KEGG [65]. First, disease genes were identified by processing all terms of gene–disease associations from the GAD. Second, for each disease, the corresponding statistically significantly enriched subpathways were identified using the “k-clique” subpathway identification method by setting k = 3 and P-value<0.01 [17]. There were 545 nodes (302 subpathways and 243 diseases) in the DMSPN. All data on the network including figures and tab delimited/excel table format are available from http://www.plosone.org/article/info%3Adoi%2F10.1371%2Fjournal.pone.0021131.

### SIDER Database

Drug side effects data were obtained from the side effect resource (SIDER), which is a public computer-readable side effect resource. The SIDER database contains 888 drugs corresponding to 1450 side effect terms. We downloaded the data file “costart_adverse_effects.tsv” from the website (http://sideeffects.embl.de) which is freely available for academic research. In the DRSN, 199 drugs were recorded in the SIDER database.

### Housekeeping Genes and Tissue-specific Genes

Housekeeping genes (HKGs) are constitutively expressed in all tissues to maintain cell functions, while tissue-specific genes (TEGs) are expressed at a much higher level in a single tissue. We downloaded the HKGs and TEGs dataset from the paper by She et al. The dataset contains 1,522 HKGs and 975 TEGs which were systematically identified from the gene expression profiles of 42 normal human tissues using conservative identification criteria [67].

### Tissue Homogeneity (TH) Coefficient

Goh et al. used the TH coefficient to quantify whether genes belonging to one disease tended to be expressed in the same tissue [69]. In our article, the TH coefficient was used to quantify whether the drug-affected TEGs in one metabolic subpathway tended to be expressed in the same tissue. Here, we defined the TH of the *i*th metabolic subpathway as follows:
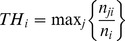
where 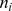 denotes the number of drug-affected TEGs in the *i*th subpathway, 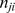 denotes the number of genes that are expressed in the tissue *j* among all the drug-affected TEGs in the ith subpathway, and 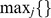 denotes the function returning the maximum-value argument across *j*. The TH coefficient has a maximal value of 1 if all the drug-affected TEGs in a subpathway are expressed together in one tissue, and has a minimum value of 0 when there are no TEGs in the drug-affected genes in a subpathway.

### Association Scores (AS) between Drugs and Diseases Through Shared Metabolic Subpathways

If a subpathway is related to some diseases and some drugs also affect this subpathway, these drugs and diseases are considered to be associated. Drug–metabolic subpathway associations and disease–metabolic subpathway associations were obtained from the DRSN and DMSPN, respectively. Then, we calculated the AS to quantify the extent of the association between any drug class and disease class using the shared subpathways. We defined the AS as follows:
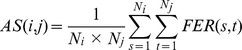



Where 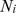 denotes the number of drugs in the *i*th drug class and 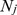 denotes the number of diseases in the *j*th disease class. The FER (fold enrichment ratio) is used to quantify the extent of the association between any drug *s* in the *i*th drug class and any disease *t* in the *j*th disease class. It is defined as 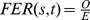, O is the observed value and E is the expected value [81]. We also use hypergeometric test to assess the overlap of the two subpathway sets of the drug *i* and disease *j*. The significant drug-disease pairs (P-value <0.01), the drug and disease names, their fold enrichment ratio and corresponding P-value are documented in Dataset S4. In our article, the observed value and expected value are identified as 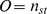 and 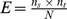 respectively, where 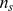 is the number of subpathways affected by drug *s*, 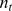 is the number of subpathways related to disease *t* and 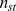 is the number of subpathways shared by drug *s* and disease *t*; 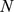 is the number of unions of subpathways which belong to the *i*th drug class or the *j*th disease class. The higher the AS value the closer the correlation between a drug class and a disease class. The AS could quantify the extent of the association between any drug class and disease class.

## Supporting Information

Figure S1 **The basic network features of the DRSN.** To estimate the background distribution of the drug–metabolic subpathway network, we randomly shuffled the drug–gene associations, while both the number of genes that a drug affected and the number of drugs that a gene was affected by remain unchanged. We generated 1000 independent randomized samples. (A) The average degree of subpathway nodes in the DRSN was significantly higher than that of 1000 randomized networks (P-value<0.001). (B) The average degree of drug nodes in the DRSN was significantly higher than that of 1000 randomized networks (P-value<0.001). (C) The number of edges in the DRSN was significantly higher than that in randomized networks (P-value = 0).
(TIF)

Figure S2 **The degree distribution of the DRSN. (**A) Degree distribution of drugs in the DRSN. (B) Degree distribution of subpathways in the DRSN.
(TIF)

Figure S3 **The relationship between drug dual effects and metabolic subpathways in the DRSN with k = 4.**
(TIF)

Figure S4 **Tissue-specific differences between therapeutic and non-therapeutic subpathways in the DRSN with k = 4.**
(TIF)

Table S1 **Literatures about some drug–subpathway associations from various significant levels**
(DOC)

Text S1. (DOC)

Dataset S1 **913 small molecule drug information.**
(XLS)

Dataset S2 **The drug-metabolic subpathway associations.**
(XLS)

Dataset S3 **Drug-Target associations.**
(XLS)

Dataset S4 **The drug–disease association through metabolic subpathways.**
(XLS)

Dataset S5 **Drug degree rank of two networks.**
(XLS)
