# Supplementary figures and images for: Characterizing the Network of Drugs and Their Affected Metabolic Subpathways
Source: PLoS One. 2012 Oct 24;7(10):e47326. doi: 10.1371/journal.pone.0047326 (PMC3480395; doi:10.1371/journal.pone.0047326)

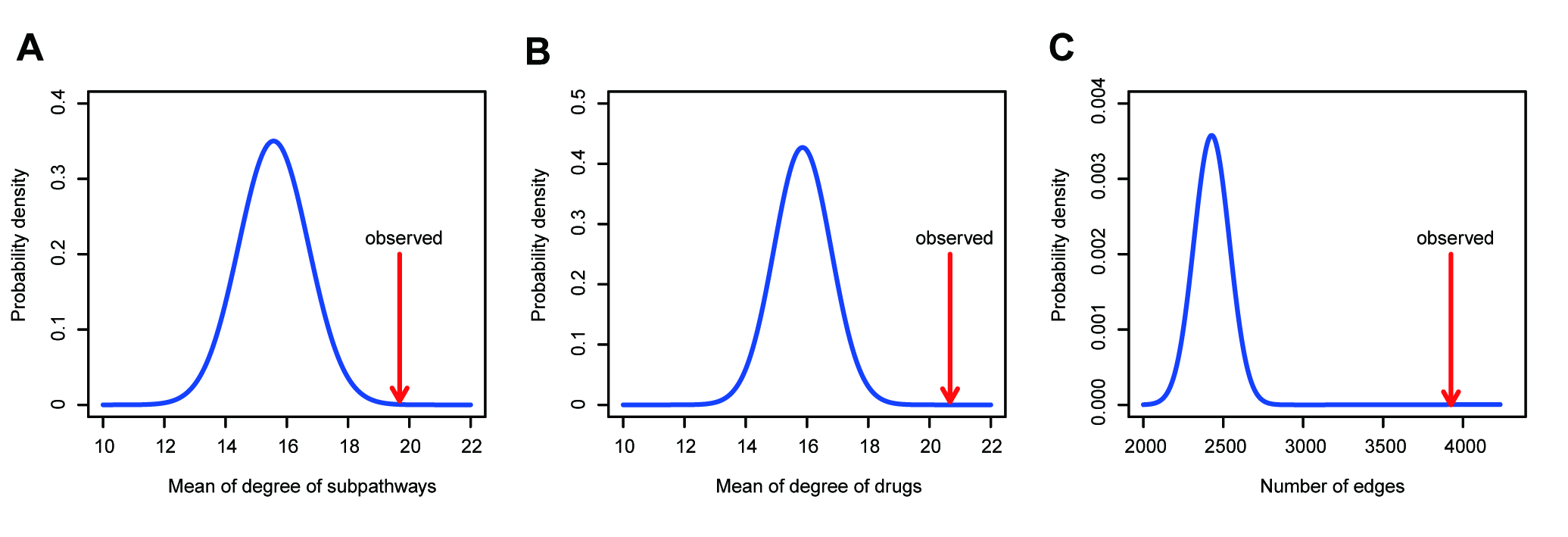

Supplement: Figure S1 — The basic network features of the DRSN. To estimate the background distribution of the drug–metabolic subpathway network, we randomly shuffled the drug–gene associations, while both the number of genes that a drug affected and the number of drugs that a gene was affected by remain unchanged. We generated 1000 independent randomized samples. (A) The average degree of subpathway nodes in the DRSN was significantly higher than that of 1000 randomized networks (P-value<0.001). (B) The average degree of drug nodes in the DRSN was significantly higher than that of 1000 randomized networks (P-value<0.001). (C) The number of edges in the DRSN was significantly higher than that in randomized networks (P-value = 0). (TIF) [file pone.0047326.s001.tif]

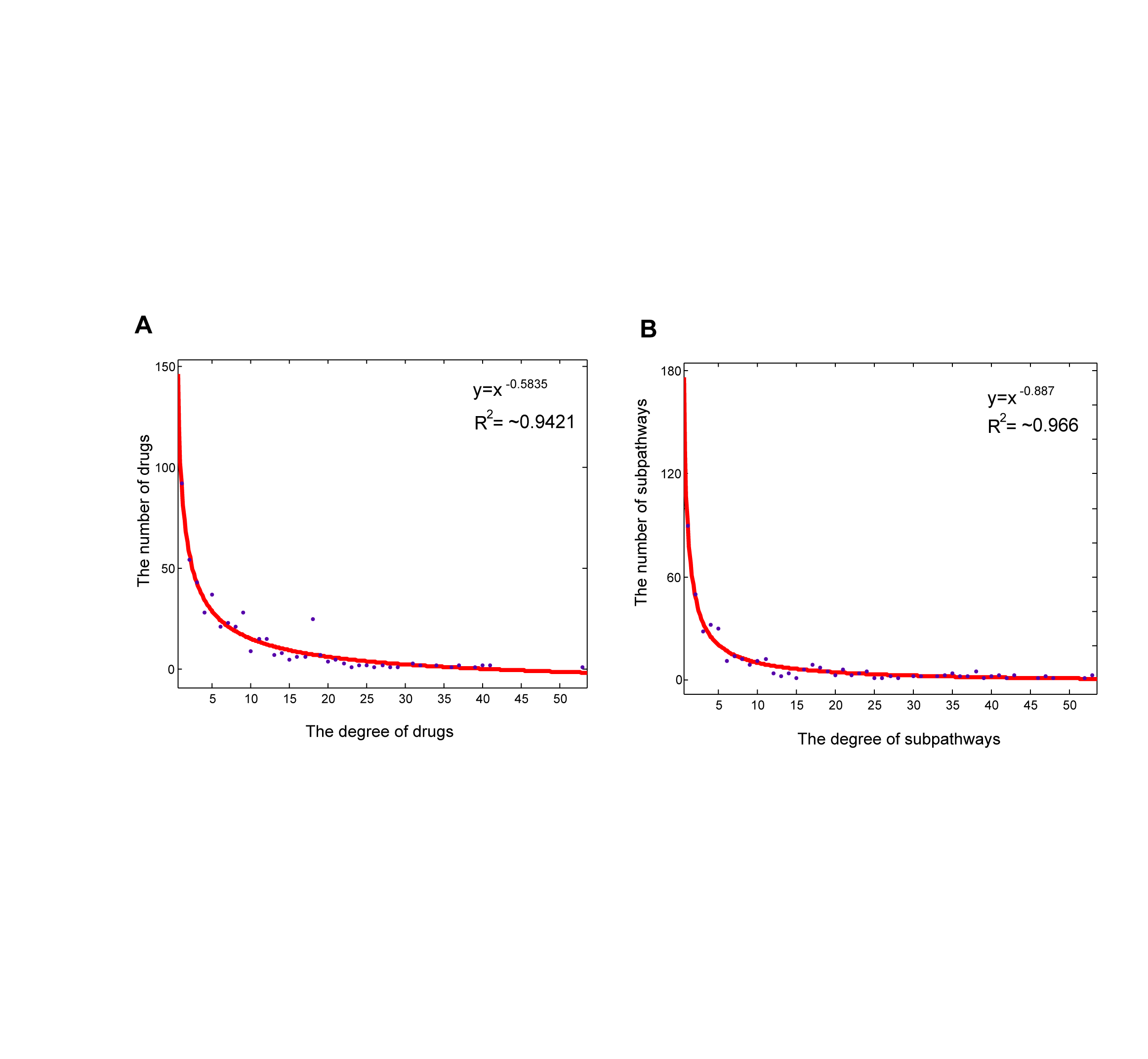

Supplement: Figure S2 — The degree distribution of the DRSN. (A) Degree distribution of drugs in the DRSN. (B) Degree distribution of subpathways in the DRSN. (TIF) [file pone.0047326.s002.tif]

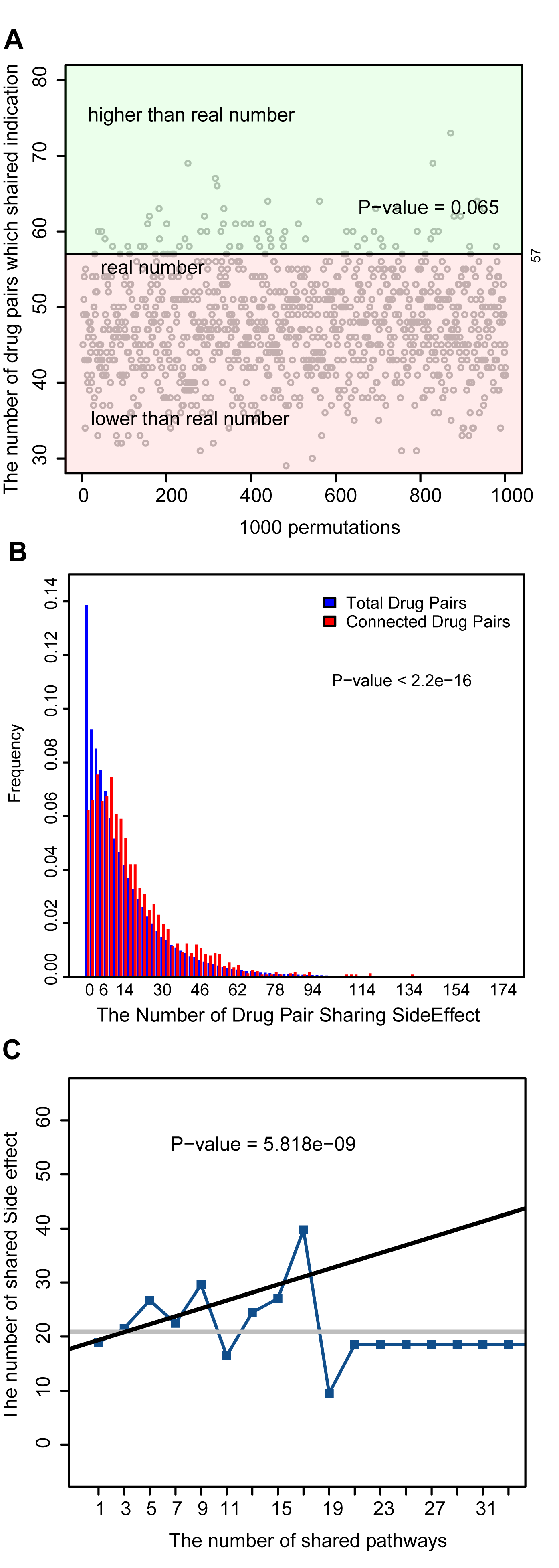

Supplement: Figure S3 — The relationship between drug dual effects and metabolic subpathways in the DRSN with k = 4. (TIF) [file pone.0047326.s003.tif]

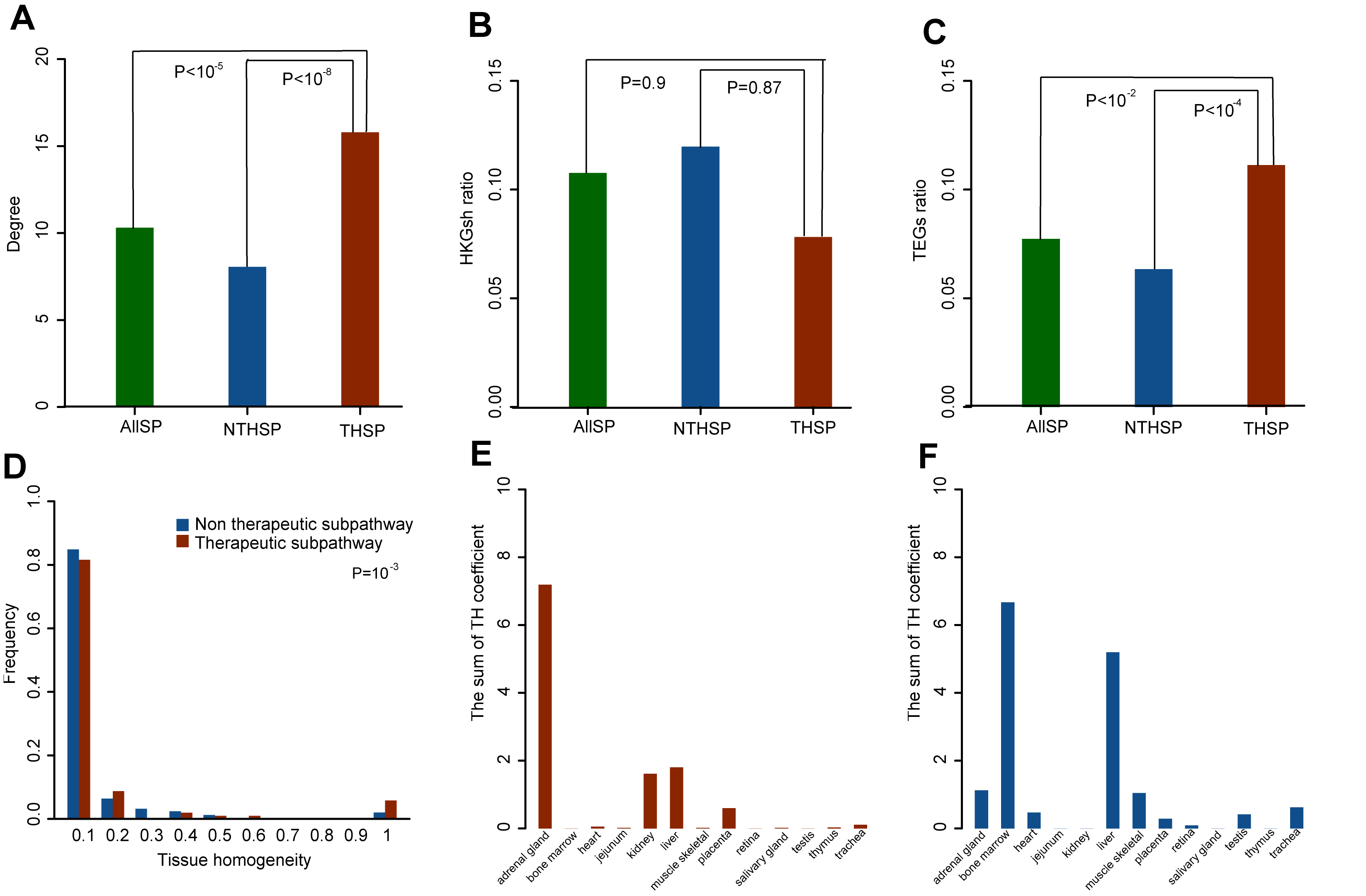

Supplement: Figure S4 — Tissue-specific differences between therapeutic and non-therapeutic subpathways in the DRSN with k = 4. (TIF) [file pone.0047326.s004.tif]
