# Supplementary material for: Characterizing the Network of Drugs and Their Affected Metabolic Subpathways
Source: PLoS One. 2012 Oct 24;7(10):e47326. doi: 10.1371/journal.pone.0047326 (PMC3480395; doi:10.1371/journal.pone.0047326)
Supplement: Table S1 — Literatures about some drug–subpathway associations from various significant levels (DOC) [file pone.0047326.s005.doc]

**Supplementary Table 1 literatures about some drug–subpathway associations from various significant levels**

| Drug | Subpathways | Significant level | Literature |
| --- | --- | --- | --- |
| yohimbine | Caffeine metabolism (path:00232_1) | 9.56e-06 | [1,2] |
| yohimbine | Tryptophan metabolism (path:00380_2) | 5.10e-05 | [3] |
| yohimbine | Androgen and estrogen metabolism (path:00150_1) | 0.0001 | [4] |
| tetracycline | Tryptophan metabolism (path:00380_2) | 2.88e-06 | [5] |
| tetracycline | Caffeine metabolism (path:00232_1) | 7.13e-05 | [6] |
| tetracycline | Fatty acid metabolism (path:00071_2) | 0.0001 | [7,8] |
| tetracycline | Arachidonic acid metabolism (path:00590_4) | 0.000367 | [9,10] |
| testosterone | Tryptophan metabolism (path:00380_3) | 0.0003165 | [11] |
| testosterone | Caffeine metabolism (path:00232_1) | 0.00152 | [12] |
| testosterone | Arachidonic acid metabolism (path:00590_3) | 0.0054 | [13] |
| testosterone | Androgen and estrogen metabolism (path:00150_2) | 0.000913 | [14,15] |
| sulindac | Arachidonic acid metabolism (path:00590_1) | 0.000381 | [16,17] |
| risperidone | Tryptophan metabolism (path:00380_1) | 8.48e-05 | [18,19] |
| riboflavin | Caffeine metabolism (path:00232_2) | 1.60e-06 | [20,21] |
| orlistat | Biosynthesis of steroids (path:00100_1) | 1.06e-05 | [22,23] |
| leflunomide | Arachidonic acid metabolism (path:00590_4) | 0.000209 | [24] |
| apomorphine | Arachidonic acid metabolism (path:00590_6) | 0.00213 | [25] |
| berberine | Tyrosine metabolism (path:00350_16) | 0.0024 | [26-29] |
| carmustine | Tryptophan metabolism (path:00380_14) | 0.00423 | [30,31] |
| cefepime | Lipopolysaccharide biosynthesis (path:00540_3) | 0.000179 | [32] |

**Supplementary references**

1. Haller C, Kearney T, Bent S, Ko R, Benowitz N, et al. (2008) Dietary supplement adverse events: report of a one-year poison center surveillance project. J Med Toxicol 4: 84-92.

2. Ventola CL (2010) Current Issues Regarding Complementary and Alternative Medicine (CAM) in the United States: Part 2: Regulatory and Safety Concerns and Proposed Governmental Policy Changes with Respect to Dietary Supplements. P T 35: 514-522.

3. Maron E, Shlik J, Nutt DJ (2008) Tryptophan research in panic disorder. Int J Tryptophan Res 1: 3-12.

4. Gajewski Z, Faundez R, Thun R, Pawlinski B (2006) Adrenergic stimulation and blocking of hormonal secretion activity of cultured cow granulosa cells. J Physiol Pharmacol 57 Suppl 8: 125-137.

5. Schmidt SK, Muller A, Heseler K, Woite C, Spekker K, et al. (2009) Antimicrobial and immunoregulatory properties of human tryptophan 2,3-dioxygenase. Eur J Immunol 39: 2755-2764.

6. Gummadi SN, Bhavya B, Ashok N (2012) Physiology, biochemistry and possible applications of microbial caffeine degradation. Appl Microbiol Biotechnol 93: 545-554.

7. Oliveira PJ, Carvalho RA, Portincasa P, Bonfrate L, Sardao VA (2012) Fatty Acid Oxidation and Cardiovascular Risk during Menopause: A Mitochondrial Connection? J Lipids 2012: 365798.

8. Federici TJ (2011) The non-antibiotic properties of tetracyclines: clinical potential in ophthalmic disease. Pharmacol Res 64: 614-623.

9. Kim GK, Del Rosso JQ (2010) Drug-provoked psoriasis: is it drug induced or drug aggravated?: understanding pathophysiology and clinical relevance. J Clin Aesthet Dermatol 3: 32-38.

10. Bastos LF, de Oliveira AC, Watkins LR, Moraes MF, Coelho MM (2012) Tetracyclines and pain. Naunyn Schmiedebergs Arch Pharmacol 385: 225-241.

11. Giltay EJ, Bunck MC, Gooren LJ, Zitman FG, Diamant M, et al. (2008) Effects of sex steroids on the neurotransmitter-specific aromatic amino acids phenylalanine, tyrosine, and tryptophan in transsexual subjects. Neuroendocrinology 88: 103-110.

12. Yeap BB, Almeida OP, Hyde Z, Norman PE, Chubb SA, et al. (2009) Healthier lifestyle predicts higher circulating testosterone in older men: the Health In Men Study. Clin Endocrinol (Oxf) 70: 455-463.

13. Vasudevan H, Yuen VG, McNeill JH (2012) Testosterone-dependent increase in blood pressure is mediated by elevated Cyp4A expression in fructose-fed rats. Mol Cell Biochem 359: 409-418.

14. Wu MV, Manoli DS, Fraser EJ, Coats JK, Tollkuhn J, et al. (2009) Estrogen masculinizes neural pathways and sex-specific behaviors. Cell 139: 61-72.

15. Rosario ER, Chang L, Beckett TL, Carroll JC, Paul Murphy M, et al. (2009) Age-related changes in serum and brain levels of androgens in male Brown Norway rats. Neuroreport 20: 1534-1537.

16. Steinbrink SD, Pergola C, Buhring U, George S, Metzner J, et al. (2010) Sulindac sulfide suppresses 5-lipoxygenase at clinically relevant concentrations. Cell Mol Life Sci 67: 797-806.

17. Marnett LJ (2009) Mechanisms of cyclooxygenase-2 inhibition and cardiovascular side effects: the plot thickens. Cancer Prev Res (Phila) 2: 288-290.

18. Batool F, Hasnat A, Haleem MA, Haleem DJ (2010) Dose-related effects of clozapine and risperidone on the pattern of brain regional serotonin and dopamine metabolism and on tests related to extrapyramidal functions in rats. Acta Pharm 60: 129-140.

19. Scheiber J, Chen B, Milik M, Sukuru SC, Bender A, et al. (2009) Gaining insight into off-target mediated effects of drug candidates with a comprehensive systems chemical biology analysis. J Chem Inf Model 49: 308-317.

20. Cui Y (2010) Parallel stacking of caffeine with riboflavin in aqueous solutions: the potential mechanism for hydrotropic solubilization of riboflavin. Int J Pharm 397: 36-43.

21. Ahmad I, Ahmed S, Sheraz MA, Aminuddin M, Vaid FH (2009) Effect of caffeine complexation on the photolysis of riboflavin in aqueous solution: a kinetic study. Chem Pharm Bull (Tokyo) 57: 1363-1370.

22. Anagnostis P, Selalmatzidou D, Sapranidis M, Panagiotou A, Polyzos SA, et al. (2012) Comparative effects of sibutramine and orlistat on weight loss, glucose metabolism and leptin levels in non-diabetic obese patients: A prospective study. Indian J Endocrinol Metab 16: 146-147.

23. Derosa G, Cicero AF, D'Angelo A, Fogari E, Maffioli P (2012) Effects of 1-year orlistat treatment compared to placebo on insulin resistance parameters in patients with type 2 diabetes. J Clin Pharm Ther 37: 187-195.

24. Yildiz Y, Kose H, Cecen S, Ergin K, Demir EM, et al. (2010) Protective effects of leflunomide on intestinal ischemia-reperfusion injury: leflunomide against intestinal ischemia-reperfusion. Dig Dis Sci 55: 245-252.

25. Bhattacharjee AK, Chang L, White L, Bazinet RP, Rapoport SI (2008) Imaging apomorphine stimulation of brain arachidonic acid signaling via D2-like receptors in unanesthetized rats. Psychopharmacology (Berl) 197: 557-566.

26. Amasheh M, Fromm A, Krug SM, Amasheh S, Andres S, et al. (2010) TNFalpha-induced and berberine-antagonized tight junction barrier impairment via tyrosine kinase, Akt and NFkappaB signaling. J Cell Sci 123: 4145-4155.

27. Kwon IH, Choi HS, Shin KS, Lee BK, Lee CK, et al. (2010) Effects of berberine on 6-hydroxydopamine-induced neurotoxicity in PC12 cells and a rat model of Parkinson's disease. Neurosci Lett 486: 29-33.

28. Al-masri IM, Mohammad MK, Tahaa MO (2009) Inhibition of dipeptidyl peptidase IV (DPP IV) is one of the mechanisms explaining the hypoglycemic effect of berberine. J Enzyme Inhib Med Chem 24: 1061-1066.

29. Xing LJ, Zhang L, Liu T, Hua YQ, Zheng PY, et al. (2011) Berberine reducing insulin resistance by up-regulating IRS-2 mRNA expression in nonalcoholic fatty liver disease (NAFLD) rat liver. Eur J Pharmacol 668: 467-471.

30. Stankiewicz-Kranc A, Bielawska A, Bielawski K, Skrzydlewska E (2009) Proline analogue of nitrosourea as a new cytotoxic prodrug. Arch Pharm (Weinheim) 342: 632-639.

31. Stankiewicz-Kranc A, Miltyk W, Skrzydlewska E (2010) Comparison of influence of carmustine and new proline analog of nitrosourea on antioxidant system in breast carcinoma cells (MCF-7). Drug Chem Toxicol 33: 55-63.

32. Pawar YG, Sharma SK (2008) Influence of E. coli lipopolysaccharide induced fever on the plasma kinetics of cefepime in cross-bred calves. Vet Res Commun 32: 123-130.
