# Supplementary material for: Characterizing the Network of Drugs and Their Affected Metabolic Subpathways
Source: PLoS One. 2012 Oct 24;7(10):e47326. doi: 10.1371/journal.pone.0047326 (PMC3480395; doi:10.1371/journal.pone.0047326)
Supplement: Text S1. — (DOC) [file pone.0047326.s006.doc]

Supplementary information

**1. Drug database**

The drug information including ATC code, drug targets and drug description were obtained from DrugBank database (Version: **2.5**) and the KEGG DRUG databases. The DrugBank database is a unique bioinformatics and cheminformatics resource that combines detailed drug data with corresponding drug target and treatment indication information [1]. In version 2.5, the database contains nearly 4800 drug entries, including >1,350 FDA-approved small molecule drugs, 123 FDA-approved biotech drugs, 71 nutraceuticals and more than 3243 experimental drugs. We downloaded all small molecular drugs. Similarly, we downloaded the drug card from KEGG (<ftp://ftp.genome.jp/pub/kegg/medicus/drug/>) [2], and extracted all the drugs which belong to small molecule. Of all 1309 small molecules in CMap, 581 small molecules were recorded as drugs in DrugBank. Of remnant 728 small molecules, 332 small molecules were recorded as drugs in KEGG DRUG. Some drugs were recorded by different names in CMap and drug database. For example, small molecule “paracetamol” was recorded as “acetaminophen” in the DrugBank small molecule “benzylpenicillin” was recorded as “penicillin g” in the DrugBank. Thus, we unified the different names of same drug into small molecular name recorded in CMap. Totally, we obtained 913 small molecular drugs (see SI Dataset S1). We used ATC code (Anatomical Therapeutic Chemical http://www.whocc.no/) for drug classification, which could be obtained from above two databases. There were still 128 drugs which had no ATC codes in 913 drugs. We manually identified ATC codes for these drugs according to their main indications by their description in literatures and above two databases.

Drug therapeutic targets were used to classify therapeutic subpathway and non-therapeutic subpathway. We extracted these therapeutic targets from DrugBank and KEGG Drug database. In the DrugBank, the drug therapeutic targets could be extracted automatically in the website. In KEGG drug card, we extracted these target using Perl and R. Some targets were recorded as KEGG Orthology (KO). We converted these KOs to Entrez Gene IDs. 4447 drugs had targets in two databases. We found that of 488 drugs in the DRSN, 293 drugs had 325 therapeutic targets (see SI Dataset S3).

**2. The Connectivity Map database**

The Connectivity Map (CMap) is a library of genome-wide transcriptional expression data from cultured human cells treated with 1309 bioactive small molecules [3]. In build 02, CMap contains more than 7000 gene expression profiles of 5 cell lines (HL60, MCF7, PC3, SKMEL5, and ssMCF7) treated with 1309 distinct bioactive small molecules. We downloaded all the gene expression profiles and its corresponding annotation file ‘‘cmap_instance_02.xls’’ from the CMap website (<http://www.broadinstitute.org/cmap/>). In the annotation file, each instance contains 15 kinds of description informations, including the instance_id, the name of small molecules, array platform, the perturbation_id and the vehicle_id, et al.. According to the perturbation_id and the vehicle_id, for each instance, we found out the corresponding treatment and control gene expression profiles. Then we used fold-change analysis to identify differentially expressed genes (DEGs) for every instance with | log2 fold change|>1. The DEGs were merged if the corresponding instances (experiments) belonged to the same drug and these genes were considered as affected genes for this drug. After above steps, we obtained 128734 unique gene–drug associations composed of 10412 drug-affected genes and 913 drugs.

**3. The SubpathwayMiner software package**

To construct drug–metabolic subpathway network (DRSN), we used the “k-cliques” subpathway identification method provided by the SubpathwayMiner software package [4] to identify statistically significantly enriched drug-related subpathways. This software package can transform the pathway structure data with KGML format provided by KEGG to R graph objects. The input of SubpathwayMiner is a gene set (in our paper, the gene set is the drug affected genes) and distance parameter k. This software can mine each metabolic subpathways and then identify statistically significantly enriched subpathways. There are 743 subpathways when k=3, and this parameter setting means that the distance among enzymes in one subpathway is not greater than 3. For each drug affected gene set, we used this software to identify significantly enriched metabolic subpathways with a P-value<0.01. Finally, we mapped 403 among the total of 743 subpathways to 488 among the total of 913 drugs and generated 3925 significant drug–subpathway associations.

**4. Disease**–**metabolic subpathway network (DMSPN)**

Recently, we constructed a disease–metabolic subpathway network according to disease genes from Genetic Association Database (GAD) and pathway structure data from KEGG [5]. The nodes in this network represent diseases or subpathways and two nodes are connected by an edge if genes of the disease are significantly enriched to the subpathway. The DMSPN is composed of 545 nodes (302 subpathways and 243 diseases), and 4288 edges. The disease related subpathways were also identified by the SubpathwayMiner with setting k=3. Of 743 metabolic subpathways with setting k=3, 302 subpathways are related to diseases and 403 subpathways are affected by drugs. There are 230 subpathways which are related to both drugs and diseases. We used these subpathways to calculate the association score to quantify the extent of association between any drug class and disease class.

**Supplementary references**

1. Wishart DS, Knox C, Guo AC, Cheng D, Shrivastava S, et al. (2008) DrugBank: a knowledgebase for drugs, drug actions and drug targets. Nucleic Acids Res 36: D901-906.

2. Kanehisa M, Goto S, Hattori M, Aoki-Kinoshita KF, Itoh M, et al. (2006) From genomics to chemical genomics: new developments in KEGG. Nucleic Acids Res 34: D354-357.

3. Lamb J, Crawford ED, Peck D, Modell JW, Blat IC, et al. (2006) The Connectivity Map: using gene-expression signatures to connect small molecules, genes, and disease. Science 313: 1929-1935.

4. Li C, Li X, Miao Y, Wang Q, Jiang W, et al. (2009) SubpathwayMiner: a software package for flexible identification of pathways. Nucleic Acids Res 37: e131.

5. Li X, Li C, Shang D, Li J, Han J, et al. (2011) The Implications of Relationships between Human Diseases and Metabolic Subpathways. PLoS One 6: e21131.
